# Reorganization of brain networks in aging: a review of functional connectivity studies

**DOI:** 10.3389/fpsyg.2015.00663

**Published:** 2015-05-21

**Authors:** Roser Sala-Llonch, David Bartrés-Faz, Carme Junqué

**Affiliations:** Department of Psychiatry and Clinical Psychobiology, University of Barcelona, Barcelona, Spain

**Keywords:** fMRI, brain networks, aging, memory, connectivity, independent component analysis, default mode network

## Abstract

Healthy aging (HA) is associated with certain declines in cognitive functions, even in individuals that are free of any process of degenerative illness. Functional magnetic resonance imaging (fMRI) has been widely used in order to link this age-related cognitive decline with patterns of altered brain function. A consistent finding in the fMRI literature is that healthy old adults present higher activity levels in some brain regions during the performance of cognitive tasks. This finding is usually interpreted as a compensatory mechanism. More recent approaches have focused on the study of functional connectivity, mainly derived from resting state fMRI, and have concluded that the higher levels of activity coexist with disrupted connectivity. In this review, we aim to provide a state-of-the-art description of the usefulness and the interpretations of functional brain connectivity in the context of HA. We first give a background that includes some basic aspects and methodological issues regarding functional connectivity. We summarize the main findings and the cognitive models that have been derived from task-activity studies, and we then review the findings provided by resting-state functional connectivity in HA. Finally, we suggest some future directions in this field of research. A common finding of the studies included is that older subjects present reduced functional connectivity compared to young adults. This reduced connectivity affects the main brain networks and explains age-related cognitive alterations. Remarkably, the default mode network appears as a highly compromised system in HA. Overall, the scenario given by both activity and connectivity studies also suggests that the trajectory of changes during task may differ from those observed during resting-state. We propose that the use of complex modeling approaches studying effective connectivity may help to understand context-dependent functional reorganizations in the aging process.

## Introduction

For many years, studies of human brain function typically associated specific cognitive domains to discrete brain anatomical structures. The evidences of brain-behavior relationships mainly emerged from studies on the consequences of focal lesions on the loss of specific cognitive functions. More recently, and mostly thanks to magnetic resonance imaging (MRI), the neuroscientific community has moved to the idea that the majority of functions are supported by coordinated activity between distinct, separated brain regions, so that the brain works in networks. These ideas have lead to the definition of *Brain Connectivity* (Catani et al., 2013; Sporns, 2013b), and *Connectomics* (Smith et al., 2013).

Brain connectivity refers to patterns of links connecting distinct units within the nervous system. It can be studied at different scales, and therefore, units or nodes can be defined as individual neurons, neural populations, or segregated brain regions, described by anatomical or functional landmarks. Recent advances in “*in vivo*” neuroimaging techniques allow the measurement of connectomics in a non-invasive way (Behrens and Sporns, 2012). In addition, the progress made on both neuroscience and computational sciences has motivated new approaches for studying brain structure and function from a complex systems perspective (Sporns, 2013a). These current trends have suggested that connectivity-based methods may provide good tools in order to understand brain functioning in healthy subjects, as well as to study changes during lifespan, or during the timecourse of neurodegenerative diseases.

In general terms, in neuroimaging, human brain connectivity can be studied at the structural and functional levels. By one hand, brain structural connectivity refers to the presence of fiber tracts directly connecting different brain regions (Basser et al., 1994). The use of *Diffusion MRI* allows investigating structural connections in the brain’s white matter by estimating the directionality of white matter fibers. On the other hand, brain functional connectivity refers to the temporal synchrony of brain activity at different regions, and it can be measured using functional MRI (fMRI).

The focus of the present review is functional connectivity in healthy aging (HA), and therefore, the results of structural connectivity studies will not be included. In this regard, it should be mentioned that the relationship between structural and functional connectivity is not always straightforward. It has been suggested that whereas functional connectivity depends on structural connectivity, structural connectivity is not sufficient to predict functional connectivity patterns (Friston, 2011). This statement should be understood under the idea that a single brain structure may support a wide variety of functions, and that functional networks usually have context-dependent or time-dependent characteristics (Park and Friston, 2013).

Furthermore, within the general term of functional connectivity, it is possible to differentiate between functional and effective connectivity. Functional connectivity aims to describe statistical dependence between measurements of neuronal activity, whereas effective connectivity refers to more complex approaches that measure the causal influence of one neural system over another. Effective connectivity is highly dependent on the context and the system dynamics, and its derived methods usually search for directionality and information flow (Friston, 2011). Here, we will first review the main findings as regards functional connectivity in aging and we will then discuss how the new approaches focused on effective connectivity may help understanding functional changes in the aging brain. In Table 1, we provide a glossary of the main terms related with connectivity within the field of neuroimaging.

**Table 1:** **Glossary of neuroimaging definitions**.

**Term**	**Definition**
Association matrix	Matrix containing the connectivity of all possible pairs of nodes in a network.
Blood-oxigen level dependent (BOLD)	MRI-related signal that measures the hemodynamic response process in the brain. It is based on the different magnetic susceptibility between oxygenated and deoxygenated blood.
Brain atlas	Structured representation of the brain in parcels. The definition of parcellations can be derived from anatomical or functional data.
Connectomics	Field within neuroscience that aims to study the brain by estimating the connections between brain regions.
Clustering	Measure of the cliquishness of connections between nodes from a topological point of view. Measures the number of triangles around a node.
Data-driven analysis	The set of techniques used to obtain patterns that exist in the data regardless of the model.
Default mode network (DMN)	Set of brain regions that are active during resting-state and that deactivate during the performance of goal-directed tasks.
Diffusion tensor imaging (DTI)	MRI modality that measures random motion of molecules. In brain’s white matter is used to estimate the direction of the fibers and to track the major fiber bundles.
Dynamic causal modeling (DCM)	Technique that estimates states and parameters of effective connectivity using observed data underlying biological or physical quantities. Used with fMRI data using Bayesian techniques.
Effective connectivity	Measurement of the causal connectivity and its directionality between brain regions. It measures information flow.
Functional connectivity	As a generic term, it refers to any pattern of connectivity obtained with functional data. More specifically, and compared with effective connectivity, it refers to the measurement of any functional connection between regions, direct or indirect, as the statistical dependence between timeseries.
Functional integration	Coordinated activity of different brain units.
Functional MRI (fMRI)	Sequential acquisition of T2*-weighted MRI volumes during the time-couse of a task or a set of events.
Functional segregation	Existence of specialized neurons and brain units that selectively respond to specific stimuli.
Granger causality analysis (GCA)	Estimation of effective connectivity between activated brain areas using vector autoregression of fMRI timeseries.
Graph	A model of a complex system, of any nature, defined by a set of nodes and the edges between them.
Independent component analysis (ICA)	A data-driven method used to obtain patterns of spatio-temporal independent processes in the data.
Model-driven analysis	The set of techniques used to analyze fMRI data that estimate patterns of activity based on the experimental model.
Pearson correlation coefficient	Measure of the linear relationship between two variables. It is used between timeseries from different regions to estimate functional connectivity.
Positron emission tomography (PET)	Technique from nuclear functional imaging that detects pairs of gamma rays emitted indirectly by a tracer introduced into the body on a biologically active molecule.
Resting-state fMRI (rs-fMRI)	A specific fMRI acquisition that measures spontaneous temporal fluctuations in brain activity “at rest.”
Resting state functional connectivity (RSFC)	Measure of the functional connectivity estimated as the temporal synchrony between spontaneous temporal fluctuations at different brain regions.
Resting state network (RSN)	Functional brain networks most commonly estimated from rs-fMRI data.
Small-worldness	Characteristic of a network, obtained from graph-theory, with high clustering and short characteristic path length. Also defined as a network with high global and local efficiency.
Structural connectivity	Estimation of structural links between brain regions. For example, the study of white matter fiber pathways.
Structural equation modeling (SEM)	Modeling for estimating effective connectivity, where model parameters are obtained as the statistical relationship between timeseries. It uses the covariance structure of fMRI timeseries to infer steady-state coupling. It does not refer to biological or physical quantities of the data.
Topology	Properties of a network obtained considering the connectivity between nodes regardless of their physical or anatomical localization.
Tractography	Method for identifying anatomical connections in the human brain *in vivo* and non-invasively using Diffusion MRI data.

T2* indicates T2 star MRI sequence.

## Methods for the Study of Functional Connectivity with MRI

Functional magnetic resonance imaging allows measuring changes in blood-oxygen-dependent (BOLD) signal in the brain across time. In its more traditional application, *task-fMRI* has been used to identify areas of increased or decreased neuronal activity during the performance of a task (Logothetis et al., 2001; Logothetis, 2003; Raichle and Mintun, 2006).

Another popular type of fMRI is the so-called *resting-state fMRI* (rs-fMRI), which refers to the sequential acquisition of fMRI scans, of duration typically between 5 and 10 min. During this time, subjects are asked to lie down, not to fall asleep and not to think in anything particular. The potential of rs-fMRI has been used to identify temporal coherences between spontaneous fluctuations that occur during rest, measured as low-frequency oscillations of the BOLD signal (Biswal et al., 1995).

During the last years, the use of rs-fMRI to study functional connectivity has increased massively and has revealed meaningful low frequency BOLD fluctuations that are correlated across distant brain regions, allowing the study of what has been called resting state functional connectivity (RSFC). Although, the origin and interpretation of these spontaneous fluctuations are still under debate (Schölvinck et al., 2010), RSFC seems to be highly informative about both brain architecture and brain organization, and it has a high variability in humans, probably reflecting behavioral inter-individual differences (Fox et al., 2007).

The analysis of rs-fMRI connectivity covers an elevated number of methodological approaches, and this number increases day-to-day thanks to technical advances and ongoing inter-disciplinary research. Basically, it is possible to differentiate between three main methodologies: seed-based connectivity analysis, independent component analysis (ICA), and whole-brain approaches using graph-theory.

### Seed-based Connectivity

This method consists on identifying whole-brain, voxel-wise connectivity maps of areas showing correlated activity with a seed, which is a delimitated brain region (a voxel or a group of voxels) defined *a priori* with data from previous analyses, from the literature or from an atlas. Although seed-based correlation methods usually have an elevated number of confounds and they are highly dependent on the seed definition and the preprocessing applied to the data, they still represent the best approach to answer directly some questions related to connectivity. The use of these methods is the best option to find, for example, correlation patterns from a certain region when there is a strong hypothesis previously formulated, providing a straightforward interpretability (Cole et al., 2010).

### Independent Component Analysis

Independent component analysis is used to find spatio-temporal patterns of synchronized brain activity. It decomposes the data into a set of independent components (IC), where each IC is formed by a spatial map and a timeseries and is independent from the other components (Beckmann and Smith, 2004). In comparison with seed-based correlation, one of its advantages is that it does not require the specification of any a priori seeds or hypothesis. Thus it is very useful for exploratory analysis. In addition, ICA appears as a good approach to identify signals of no-interest, such as artifacts, head motion, physiological noise or CSF-related signals, which can be then easily removed from the original fMRI data (Griffanti et al., 2014).

### Graph-theory Approaches

These kind of studies aim to investigate the overall brain connectivity by describing the brain as a single interconnected network (Bullmore and Sporns, 2009). They belong to the set of higher-level models used to evaluate functional connectivity in a more integrative way than the two methods described above. Graph-theory studies require, in general, a first stage in order to parcellate the brain into a set of regions or nodes. Then, in a second stage, one would find the relationships between all possible node pairs, defining a “big” whole-brain network. Once the whole-brain network is defined, it can be studied at different levels of complexity or specificity. For example, it is possible to obtain connectivity characteristics at regional level, and it is possible to obtain parameters reflecting whole-brain organization, including efficiency, integration or segregation (Rubinov and Sporns, 2010). Furthermore, using measures of nodal connectivity or centrality it has been possible to define cortical hubs as a key-connected brain regions, that have an special role in controlling connectivity paths across the whole brain (Buckner et al., 2009; Cole et al., 2010; Power et al., 2013).

## Network Discovery in Healthy Subjects

There is an outstanding interest in understanding functional brain organization in normal or healthy brains. It appears as an essential need in order to further define neuropsychological correlates and potential clinical biomarkers for neurodegenerative diseases, giving that the majority of these diseases have been described as disconnection syndromes (Geshwing and Kaplan, 1962; O’Sullivan et al., 2001; Seeley et al., 2009). In addition, it brings new insight to the design of interventional studies and to track brain changes longitudinally.

The use of rs-fMRI to study functional connectivity has allowed the identification of a reduced set of networks or connectivity patterns named resting state networks (RSNs). These networks are commonly identified across subjects (Damoiseaux et al., 2006), and have shown high reproducibility rates (Guo et al., 2012). In addition, RSNs have been associated with networks of brain functions (Sadaghiani and Kleinschmidt, 2013).

The most-studied RSNs is the default mode network (DMN), which has the specific property of being deactivated during the performance of goal-directed tasks and shows high levels of activity at rest. It was first identified as a set of regions commonly activated during passive compared with active conditions, using positron emission tomography (PET; Shulman et al., 1997; Raichle et al., 2001) and task-fMRI (Gusnard and Raichle, 2001). The DMN was further identified in a series of resting-state functional connectivity studies (Greicius et al., 2003; Fox et al., 2005; Fransson, 2005; Damoiseaux et al., 2006; Vincent et al., 2006). By gathering together studies of task-induced deactivations and functional connectivity analyses, Buckner et al. (2008) defined the core regions associated with the brain’s default network: the ventral/dorsal medial prefrontal cortex (PFC), the posterior cingulate and retrosplenial cortex, the inferior parietal lobule and the hippocampal formation (including entorhinal cortex and parahippocampal cortex).

**FIGURE 1:**
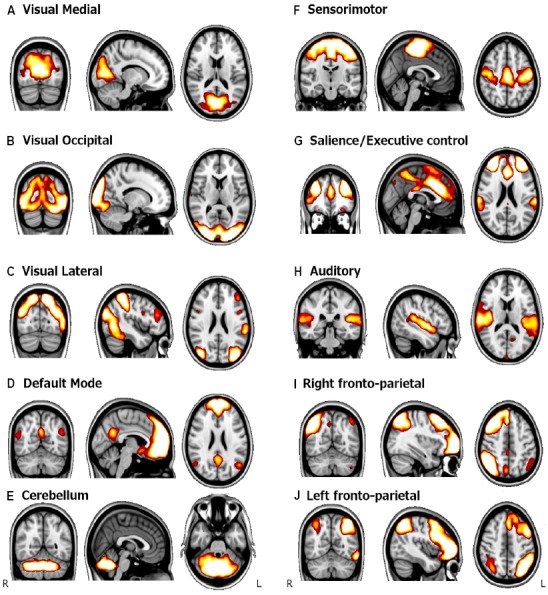
**Spatial maps of the main RSNs.** Paterns are obtained using ICA with a group of healthy young subjects. Adapted from Palacios et al. (2013). **(A)** Visual medial network, **(B)** Visual occipital network, **(C)** Visual lateral network, **(D)** Default mode network, **(E)** Cerebellum, **(F)** Sensorimotor network, **(G)** Salience network, **(H)** Auditory network, **(I)** Right fronto-parietal network, and **(J)** Left fronto-parietal.

Besides the DMN, other networks of intrinsic brain connectivity have been consistently described in healthy population. Different parcellations can be derived from resting-state functional connectivity data. For example, by using resting state data from 1000 subjects, Yeo et al. (2011), divided the human cortex into 7 and 17 networks of functionally coupled regions, with hierarchical relationship between the two parcellation schemes. Other studies have focused on the similarity between resting-state connectivity patterns, and task-based functional networks. In this regard, several studies agree in a set of 10 RSNs, covering the full repertory of task-related brain activation patterns (Fox et al., 2005; Damoiseaux et al., 2006; Smith et al., 2009). These findings indicate that the human brain has a network-based organization even at rest. In this regard, Smith et al. (2009) used ICA on rs-fMRI data and compared the components with task-patterns averaged from the *BrainMap* database (Laird et al., 2005) which assembled results from more than 7000 task-fMRI experiments. They found that the patterns of RSFC could be easily associated with patterns of task-related co-activations from a wide range of cognitive domains. The spatial maps of the 10 most commonly defined networks are illustrated in Figure 1.

Apart from the studies that have used ICA to describe the main RSNs, other researchers have focused on whole-brain approaches to investigate patterns of RSFC in healthy brains. For example, Crossley et al. (2013) used graph-theory to define a network from rs-fMRI, and they compared this network with a network of task-co-activation patterns obtained from the BrainMap Database (Laird et al., 2005). They described a brain structure based on functional connectivity patterns that showed modular organization and which was very similar between task and rest. Concretely, they defined four modules that were associated with different functions: the occipital module (perception), the central and sensorimotor module (action), the frontoparietal module (executive functions) and the DMN (emotion). The authors concluded that there is a well-defined network organization in the brain that is equally evidenced at rest and during task performance. In another study, Cole et al. (2014) studied connectivity patterns by creating whole-brain networks from data obtained at rest and while subjects performed a variety of cognitive tasks. They defined an intrinsic network structure obtained from rs-fMRI, which was highly dominant in the resting brain and even during the performance of a task. Interestingly, they also found that this network structure is slightly modulated by task-evoked connectivity changes that were both task-general and task-specific.

## FMRI in Healthy Aging

From the behavioral point of view, it is known that some adults are able to maintain their cognitive capabilities at high levels, in contrast with other persons who show clear cognitive declines with advancing age. It has been hypothesized that this variability depends on neurofunctional resources. However, the exact mechanisms that lead to such wide differences are still unclear (Park and Reuter-Lorenz, 2009).

The use of task-fMRI in aging has revealed a complex pattern of brain activity changes, which is characterized by both, decreases and increases in old subjects compared to young subjects (Grady, 2012). In some cases, the diversity of findings depends on many variables, such as the cognitive tests used and their level of difficulty (Grady et al., 2006). Nonetheless, there is a relative consensus that there is an age-related increase of brain activity in the (PFC; Turner and Spreng, 2012), while the findings as regards reduced activation are localized more heterogeneously in the brain.

In this part, we will review some of these main theories that have appeared in the attempt to explain the trajectories of brain changes and their relationship with cognition. It is important to note that whereas earlier or “more classical” views aimed to provide meaningful interpretations of a variety of isolated phenomena, such as the increased or the decreased regional brain activity in old compared with young subjects, more recent theories aim to provide a global, integrative interpretation of brain changes.

### Classical Theories Derived from Task-fMRI Studies

In general, regional hyperactivation has been interpreted as compensation (or an attempt to compensate), whereas a failure to activate or reduced activation has been typically related with cognitive deficits associated with aging. Two main hypotheses were proposed to explain the nature of these age-related activity changes: the *dedifferentiation* hypothesis and the *compensation* hypothesis.

By one hand, the term dedifferentiation is described as the loss of functional specificity in the brain regions that are engaged during the performance of a task (Park et al., 2004; Rajah and D’Esposito, 2005). In neurobiological terms, it has been suggested that this pattern of changes is caused by a chain of processes which starts from a decline in the dopaminergic neuromodulation that produces increases in neural noise, leading to less distinctive cortical representations (Li et al., 2001).

On the other hand, the compensation hypothesis in aging states that older adults are able to recruit higher levels of activity in comparison to young subjects in some brain areas to compensate for functional deficits located somewhere else in the brain. This increased activity is often seen in frontal regions (Park and Reuter-Lorenz, 2009; Turner and Spreng, 2012). The first studies suggesting compensatory mechanisms appeared early in the literature and used PET during the performance of visuospatial (Grady et al., 1994) or episodic memory (Cabeza et al., 1997; Madden et al., 1999) tasks. Later on, these findings were replicated with fMRI (Cabeza et al., 2002).

Furthermore, the different patterns of spatial localization of the compensation-related mechanisms leaded to the formulation of three main cognitive models:

(1) The *Hemispheric Asymmetry Reduction in Old Adults* (HAROLD) model (Cabeza, 2002) states that older adults use a less lateralized pattern of activity in comparison with young subjects during the performance of a task, which is compensatory. This reduced lateralization was mainly observed in frontal areas, during the performance of episodic memory and working memory tasks (Cabeza et al., 2002; Cabeza, 2004).
(2) The *Compensation-Related Utilization of Neural Circuits Hypothesis* (CRUNCH; Reuter-Lorenz and Cappell, 2008; Schneider-Garces et al., 2010) defends that, in older adults, higher neural recruitment occurs in cognitive levels that typically imply lower brain activity in younger subjects. This effect has been observed in the PFC and also in the parietal cortex, concretely in the precuneus and posterior cingulate and both in episodic memory tasks (Spaniol and Grady, 2012) and in working memory tasks (Mattay et al., 2006; Reuter-Lorenz and Cappell, 2008).
(3) The *Posterior-Anterior Shift with Aging* (PASA) was experimentally proved by Davis et al., who used two different tasks, visuoperceptive and episodic retrieval and found that older subjects had deficits to activate regions in the posterior midline cortex accompanied with increased activity in medial frontal cortex (Davis et al., 2008).

### Global, Integrative Theories of Cognitive Function and the Aging Brain

With the unique information provided by fMRI activity and with the classification described above, which presents the models as being exclusive between them, it seems difficult to discern which of the proposed model better explains the age-related changes in cognition.

More recently, an important contribution to the interpretation of these models has been given by multimodal studies that integrate structural and functional brain measures. For example, in some cases, it has been reported that reduced activity in task-related regions correlated positively with brain atrophy in the same brain regions (Brassen et al., 2009; Rajah et al., 2011), whereas other studies have reported correlations between the increased functional activity in the PFC and the preserved structural integrity of the entorhinal cortex and other medial temporal lobe (MTL) structures (Rosen et al., 2005; Braskie et al., 2009). Given this, some authors have theorized that while increased activity in the PFC may be triggered by the atrophy of frontal GM, which is a commonly reported feature in aging, the compensatory role of this increased activity may depend on the preserved structural integrity of distal regions mainly in the MTL (Maillet and Rajah, 2013).

Therefore, and mainly thanks to the new advances in neuroimaging techniques, it has been suggested that cognitive function in aging is a result of a sum of processes, including structural and functional brain measures as well as external factors. In this regard, the scaffolding theory of aging and cognition (STAC) states that there is a process in the aging brain, called compensatory scaffolding that entails the engagement of additional neural resources (in terms of network reorganization) providing a support to preserve cognitive function in the face of structural and functional decline (Park and Reuter-Lorenz, 2009). This theory has been recently revised in order to include the more recent findings on the field, obtained mainly from longitudinal and interventional studies. As a result, the STAC-r is a conceptual model that extends the STAC by incorporating life-course influences that enhance, preserve, or compromise brain status, compensatory potential and cognitive function over time (Reuter-Lorenz and Park, 2014).

In a similar sense, Walhovd et al. (2014) proposed a system-vulnerability view of cognition in aging. According to them, the age-associated cognitive decline would be the result of a life-long accumulation of impact that alters brain function and structure in a multidimensional way, affecting a wide range of neuroimage markers such as structural integrity, functional activity and connectivity, glucose metabolism, or amyloid deposition. According to this view some particular brain systems such as the hippocampus and posteromedial regions would be particularly vulnerable to ageing effects, related to its central role as mechanisms subtending lifetime brain plasticity (Fjell et al., 2014).

Finally, a complementary hypothesis, also emerged from the results of longitudinal studies is the “brain maintenance,” which states that the lack of changes in brain structural and functional markers would allow some people to show little or no age-related cognitive decline. The conceptual idea of brain maintenance was motivated by the fact that increased functional activity in HA do not necessarily imply up-regulation of functional networks over time. Therefore, according to maintenance, the best predictors of successful performance in aging would be the minimization of chemical, structural and functional changes over time (Nyberg et al., 2012).

## Connectivity-related Changes in Aging

Results from task-activation fMRI studies in aging are sometimes controversial and difficult to interpret. Therefore, more recently, studies on HA have also taken advantage from the advances as regards brain connectivity (Dennis and Thompson, 2014). Brain connectivity changes related with aging are thought to be useful in order to interpret functional reorganizations in the context of the models mentioned above of functional brain compensation and dedifferentiation.

Some evidences of task-related connectivity changes in aging are found in the working memory literature. Nagel et al. (2011) found load-related increases in PFC activity accompanied with decreases in the functional coupling between PFC and premotor cortex. Madden et al. (2010) studied task switching and found similar levels of brain activity between young an old groups, with lower functional connectivity in older subjects. Similarly, in episodic memory tasks, connectivity changes have been described (Daselaar et al., 2006; Dennis et al., 2008; Addis et al., 2010). Concretely, these studies reported reduced connectivity from the hippocampus and MTL to posterior and occipital regions together with increased connectivity from the same regions to frontal areas, such as the PFC. These results support the PASA model and indicate that functional connectivity changes follow similar patterns than those described with task-related activity.

In addition to task-fMRI studies, functional connectivity in aging has been primarily studied with rs-fMRI. Alterations of RSFC in aging include disconnection or dysfunction within some of the large-scale networks as well as alterations in whole-brain connectivity patterns. A summary of the most relevant studies in aging and functional connectivity using rs-fMRI, including those that reported correlations with cognitive changes, is given in Table 2.

**Table 2:** **Summary of functional connectivity studies in healthy aging**.

**Study**	**Sample**	**Methodology**	**RSN Changes**	**Other results**	**Relationship with cognition**
Achard and Bullmore (2007)	17 young (18–33 years) 13 old (62–76 years)	Graph-theory	–	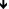 Global efficiency Localized effects in frontal and temporal regions	–
Andrews-Hanna et al. (2007)	93 (18–93 years)	Seed-based	DMN 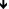 DAN 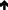	FC relates to white matter integrity	Executive functions, memory and processing speed
Meunier et al. (2009)	17 young (18–33 years) 13 old (62–76 years)	Graph-theory	–	Equal modularity 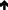 number of modules 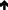 segregation	–
Wang et al. (2010)	17 (62–83 years)	Seed-based	DMN 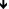	FC hippocampus-PPC	Prediction of memory performance
Jones et al. (2011)	341 (64–91 years)	ICA Seed-based	DMN 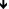 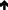	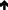 Anterior DMN FC 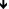 Posterior DMN FC	Correlation with mental state test
Campbell et al. (2012)	12 young (18–28 years) 12 old (60–78 years)	Seed-based	FPN 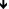 CN 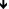	FC relates to task-activity	–
Onoda et al. (2012)	73 (36–86 years)	ICA Seed-based	SN 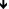 DMN 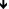	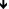 SN-Visual 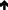 SN-Auditory 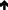 DMN-Visual	SN correlates with frontal and visuospatial functions
Tomasi and Volkow (2012)	913 (13–85 years)	FC density mapping (whole-brain).	DMN 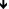 DAN 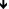 SomMotor 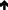 Subcortical 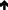	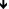 long-range FC 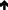 short-range FC	–
Betzel et al. (2014)	126 (7–85 years)	Whole-brain FC Graph-theory	CN 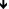 DMN 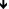 VisPeri 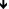 SNÙ ^ SomMotor ^ VisCen ^	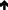 FC between RSNs	–
Geerligs et al. (2014)	40 young (18–26 years) 40 old (59–74 years)	Graph-theory	DMN 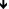 CingOper 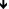 FPN 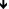 SomMotor = Visual =	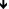 Modularity 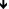 Locaf efficiency 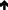 DMN–CN 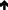 Visual–CN	–
Song et al. (2014)	26 young (24.46 ± 3 years) 24 old (58 ± 6.1 years)	Graph-theory	DMN 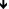 SomMotor 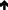	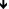 Modularity 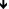 Local efficiency Change in hubness	–
Zhang et al. (2014)	18 young (22–33 years) 22 old (60–80 years)	Seed-based	DMN 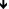 SN 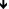 CN 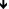 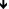 DAN 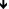 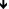 Visual =	Selective vulnerability of networks	–
Sala-Llonch et al. (2014)	98 old (64.87 ± 11.8 years)	Graph-theory	–	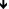 Long-range FC 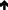 Short-range FC 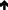 Clustering 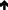 Minimum path length	Clustering correlates with verbal and visual memory function

CingOper, Cingulo-Opercular network; CN, Control Network; DAN, Dorsal Anterior Network; DMN, Default Mode Network; FC, Functional Connectivity; PPC, Precuneus/Posterior Cingulate; RSN, Resting-State Networks; SN, Salience Network; VisCen, Visual Central; VisPeri, Visual Pericalcarine; SomMotor, somatosensory/motor network; 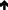

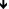
, indicates increases/decreases in connectivity; =, indicates no changes in connectivity; ^, indicates non-linear changes in connectivity.

It is noteworthy that a great majority of articles have focused on the DMN. This fact can be explained because the DMN has been related to the functional and neurobiological changes underlying Alzheimer’s Disease (AD), specially at its first stages (Buckner et al., 2009), which is the most common neurodegenerative disease affecting aged population.

A common finding of the studies reviewed in Table 2 is the decreased connectivity within the nodes of some of the main RSNs, including the DMN and the Salience and executive/attention networks. This result has been observed using ICA (Damoiseaux et al., 2008; Jones et al., 2011; Onoda et al., 2012), and also using seed-based connectivity (Andrews-Hanna et al., 2007; Wang et al., 2010) and graph-theory or whole-brain approaches (Tomasi and Volkow, 2012; Betzel et al., 2014; Geerligs et al., 2014; Song et al., 2014). Disrupted connectivity in aging persists even controlling for brain atrophy or age-related structural changes (Ferreira and Busatto, 2013). Connectivity decreases directly imply reductions in how information is transferred between different brain regions. In this regard, a commonly result is the disconnection between the anterior and the posterior nodes of the DMN, which correlates with age-related cognitive decline (Andrews-Hanna et al., 2007; Damoiseaux et al., 2008), and with white-matter alterations (Andrews-Hanna et al., 2007).

The results as regards somatosensory, motor and subcortical networks are not as consistent as with the DMN. Some studies have reported connectivity increases (Tomasi and Volkow, 2012; Song et al., 2014), no changes in connectivity (Geerligs et al., 2014) or non-linear changes (Betzel et al., 2014).

Connectivity changes have been further explored using higher-level analysis methods. Tomasi and Volkow (2012) found that long-range connectivity decreased with age whereas short-range connections were stronger. These results were interpreted under the hypothesis that some brain regions, with key roles in whole-brain connectivity, named hubs (Buckner et al., 2009; Crossley et al., 2013), could experiment strengthening of functional connectivity with their closest regions, leading to an increase in local connectivity (Ferreira and Busatto, 2013). In addition, another line of research refers to the study of functional connectivity within and between the main large-scale networks. In this regard, it has been described that the age-related decreases in connectivity between regions of a network are accompanied by increases in the connectivity of these network toward regions of other RSNs, affecting the overall functional connectivity architecture (Betzel et al., 2014; Geerligs et al., 2014).

Finally, few papers have reported relationships between connectivity and cognition. In some cases, connectivity changes have been related to executive and memory functions (Andrews-Hanna et al., 2007; Damoiseaux et al., 2008; Wang et al., 2010; Onoda et al., 2012; Sala-Llonch et al., 2014). Decreased functional connectivity has been also correlated with decreased structural connectivity in aging (Andrews-Hanna et al., 2007).

## Future Directions

Importantly, in the upcoming years, multi-centric international projects such as the Human Connectome Project (HCP, http://www.humanconnectome.org/) will represent an important contribution to understand human brain connectivity. The HCP consortium has recently presented a database for housing and disseminating publicy available human brain connectivity data (Hodge et al., in press). This database includes data from multiple MRI modalities, magnetoencephalography (MEG) data, as well as its associated cognitive and behavioral data. It currently includes data from young subjects, but additional related projects will focus on life-span trajectories of human brain connectivity. We believe that these initiatives will provide an excellent tool to test the cognitive models of the aging brain and to understand changes that are related to network reorganization processes.

It should be noted that all the studies mentioned in this review are based on the study of functional connectivity as the statistical dependence between timeseries. This definition of functional connectivity, as opposite to effective connectivity, does not allow inferring causality and it is less biologically meaningful. However, in general, functional connectivity analyses are more robust and faster to compute (Smith et al., 2013). In addition, they still represent the best and most used approach for rs-fMRI data.

On the other hand, these more complex network modeling approaches, related with effective connectivity, can measure directional and causal relationships between network nodes and they are thought to provide more biologically interpretable results (Friston, 2011). These methods were initially designed to study task-fMRI data, giving the fact that they refer to the study of how information flows among the regions of a network as a response to a specific stimulus. Several approaches have been proposed to estimate effective connectivity, including structural equation model (SEM; McLntosh and Gonzalez-Lima, 1994), granger causality analysis (GCA; Goebel et al., 2003), and dynamic causal modeling (DCM; Friston et al., 2003). Of the three, both SEM and GCA methods have shown many controversies as regards their applicability for fMRI data, and DCM seems the best approach for fMRI timeseries (Friston, 2011; Di and Biswal, 2014).

Only few studies have reported results as regards effective connectivity in aging (Addis et al., 2010; Waring et al., 2013). These studies have shown age-related modulations in networks involved in selective memory in emotional domains. For example, Waring et al. (2013) reported that older adults showed stronger connectivity during task within frontal regions and from frontal regions to MTL. Models of effective connectivity have been also applied in the context of neurodegenerative diseases, such as Alzheimer’s Disease (Rosenbaum et al., 2010; Jacobs et al., 2012) or Parkinson’s Disease (Trujillo et al., 2015).

We believe that the use of effective connectivity to study network models in aging can provide a more meaningful interpretation of the results reported so far both as regards patterns of brain activity and connectivity. For example, DCM has been used in healthy young samples to study context-dependent modulations within the fronto-parietal network (Dima et al., 2014; Harding et al., 2015). These studies described patterns reflecting the functional adaptability of different neural representations within a common system. From the set of studies included in the present review, we have concluded that increased activity during task coexists with decreased connectivity, mainly measured at rest, suggesting that age-related changes in the brain networks would have a strong context-related component. In addition, the PFC appeared as a core region related to compensatory mechanisms. Therefore, as an example, a potential application of effective connectivity models in old adults could address how functional signals from this area toward other structures is modified from resting-state acquisitions to task-related fMRI studies tapping on cognitive domains typically affected in ageing such as memory or executive functions.

However, although the use of such complex models seems promising, there are still several limitations as regards their applicability and their implementation that need to be solved. First, they are computationally sophisticated and not very robust. In addition, they were originally meant for task-fMRI data, and their adaptations for resting-state data are still on their early developments (Friston et al., 2014).

### Conflict of Interest Statement

The authors declare that the research was conducted in the absence of any commercial or financial relationships that could be construed as a potential conflict of interest.
